# Polymeric Binder Design for Sustainable Lithium-Ion Battery Chemistry

**DOI:** 10.3390/polym16020254

**Published:** 2024-01-16

**Authors:** Juhee Yoon, Jeonghun Lee, Hyemin Kim, Jihyeon Kim, Hyoung-Joon Jin

**Affiliations:** 1 Program in Environmental and Polymer Engineering, Inha University, Incheon 22212, Republic of Korea; 22231481@inha.edu (J.Y.); kimhm1124@inha.edu (H.K.); jihyunzla@inha.edu (J.K.); 2 KU-KIST Graduate School of Converging Science and Technology, Korea University, Seoul 02841, Republic of Korea; jeonghunlee@korea.ac.kr; 3 Department of Polymer Science and Engineering, Inha University, Incheon 22212, Republic of Korea

**Keywords:** Li-ion battery, polymer binders, anodes, cathodes, biopolymer, water-soluble binder, ultra-thick electrode

## Abstract

The design of binders plays a pivotal role in achieving enduring high power in lithium-ion batteries (LIBs) and extending their overall lifespan. This review underscores the indispensable characteristics that a binder must possess when utilized in LIBs, considering factors such as electrochemical, thermal, and dispersion stability, compatibility with electrolytes, solubility in solvents, mechanical properties, and conductivity. In the case of anode materials, binders with robust mechanical properties and elasticity are imperative to uphold electrode integrity, particularly in materials subjected to substantial volume changes. For cathode materials, the selection of a binder hinges on the crystal structure of the cathode material. Other vital considerations in binder design encompass cost effectiveness, adhesion, processability, and environmental friendliness. Incorporating low-cost, eco-friendly, and biodegradable polymers can significantly contribute to sustainable battery development. This review serves as an invaluable resource for comprehending the prerequisites of binder design in high-performance LIBs and offers insights into binder selection for diverse electrode materials. The findings and principles articulated in this review can be extrapolated to other advanced battery systems, charting a course for developing next-generation batteries characterized by enhanced performance and sustainability.

## 1. Introduction

The advent of lithium-ion batteries (LIBs) has ushered in innovation in mobile electronic devices, catalyzing a significant industrial transformation extending beyond the core markets of modern industry [1]. The market share and application areas of LIBs are rapidly expanding, with growing global demand for advanced LIBs characterized by high performance and sustainability, posing a current major challenge in LIB chemistry [2]. Traditionally, LIB chemistry concentrates on active electrode materials to achieve higher energy densities. However, cathode design based on layered materials is reaching its theoretical limits, and graphite anodes incorporating Si face challenges related to poor cycling stability [2,3]. These technical barriers threaten the advancement of the LIB industry, potentially leading to a recession in associated industries. Additionally, there is increasing concern regarding the nonenvironmentally friendly components in current LIB systems, particularly fluorine-based polymers, which can critically impact human health and the environment throughout their production, manufacturing, and disposal processes [4]. Despite these concerns, the binder market is largely dominated by polyvinylidene fluoride (PVdF) owing to its well-balanced material properties, required for LIBs and their fabrication processes [5]. A novel research approach has recently focused on substituting polymer binders for both the anode and cathode because of their inadequate electrochemical performance and environmental considerations [6,7].

Overcoming the theoretical capacity limitations of active cathode materials involves creating a thicker cathode, which can double the energy density of LIBs by reducing the loading contents of other inactive cell components with electrode thickening [8,9,10]. Additionally, addressing the insufficient capacity of the graphite anode (~372 mA h g^−^^1^) involves incorporating high-capacity Si, with the effectiveness of these improvements relying heavily on the performance of binder materials [11,12]. For thick cathodes, issues such as electrode cracking and flaking during the fabrication process, as well as insufficient ionic transfer rates and electrode stability during repetitive cycling, must be addressed by designing high-performance polymer binders [13,14,15,16,17]. In the case of composite anodes, the suppression of large volume expansion and the continuous formation of solid–electrolyte-interface (SEI) byproducts necessitates the introduction of functional polymer binders. Numerous case studies underscore the importance of polymer binders and their positive roles in thick cathodes and composite anodes [18].

**Figure 1:**
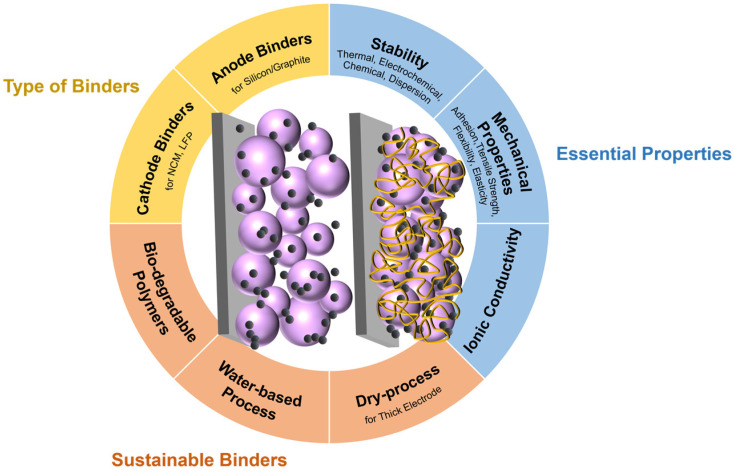
Schematic of binder design considerations in LIB.

The design of new polymer binders is also an approach towards sustainable LIB chemistry. The careful selection of an appropriate binder design plays a crucial role in maintaining a stable electrode structure even after prolonged cycles, facilitating the smooth movement of charge carriers. Despite the relatively small amount of binder used in electrodes, which is approximately 5% of the mass of commercial electrodes, its impact on the performance of energy storage devices is significant. Moreover, the conventional slurry-based electrode fabrication process, which involves the use of toxic organic solvents and generates volatile organic compounds, can be alleviated by incorporating water-soluble polymer binders or developing solvent-free binder materials based on a drying process [4,7,19,20]. In response to the environmental disadvantages associated with organic solvents, ongoing research aims to identify eco-friendly alternatives, leading to the emergence of water-solvent binders, also known as aqueous-based polymeric binders. Water-based binders, including styrene–butadiene rubber (SBR)/carboxymethyl cellulose (CMC), polyacrylic acid (PAA), chitosan, and alginates, are gaining attention owing to their eco-friendly slurry process. Furthermore, some bio-compatible and bio-degradable polymer binders exhibit high potential as functional binders for thick cathodes and composite anodes [14,15]. This review is centered on the fundamental requirements for sustainable polymer binders in both conventional electrodes and next-generation thick electrode systems in LIBs. Figure 1 illustrates types of binders and sustainable binders, including the essential properties of binders used in LIB.

## 2. Essential Properties of Binders

### 2.1. Stability

#### 2.1.1. Thermal Stability

**Figure 2:**
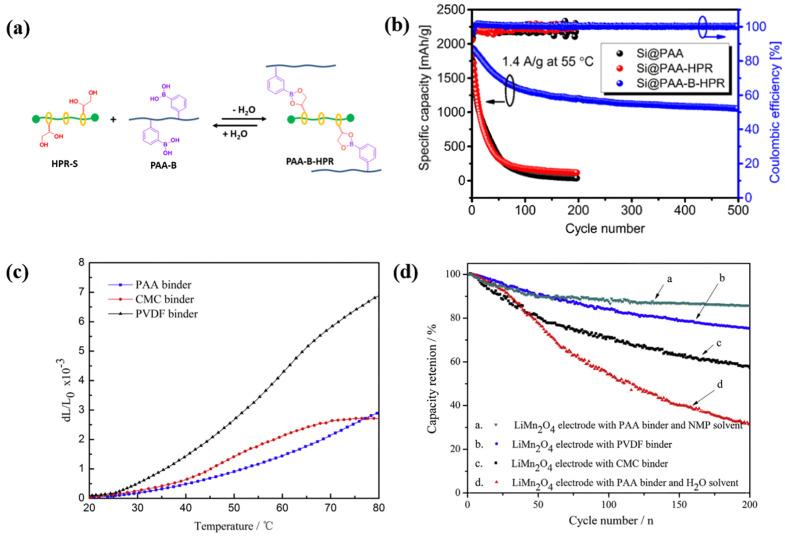
(**a**) Schematic of the synthesis of PAA cross-linked with hydroxypropylpolyrotaxanes (PAA-B-HPR). (**b**) Cycling performance of Si anodes at 1.4 A g^−1^ under 55 °C. Reprinted with permission from [23], 2021, American Chemical Society. (**c**) Thermal expansion rate curves for PAA, PVdF, and CMC binders at a temperature range of 20 to 75 °C. (**d**) Discharge cycle performances of LMO cathodes with four different binder systems at a rate of 1 C between 3 and 4.3 V at 25 °C. Reprinted with permission from [24], 2014, Elsevier B.V.

Thermal stability is a crucial property in polymer binders designed for LIBs [21]. Despite their typical operating temperature range remaining below 55 °C, polymer binders may be exposed to higher temperatures exceeding 100 °C during the fabrication process, as well as unexpected increases in their operational temperature [22]. Consequently, a high level of thermal stability is imperative across a broad temperature spectrum. Most cross-linked and cyclic polymers exhibit elevated thermal stabilities (Figure 2a,b) in contrast with conventional PVdF binders, which tend to loosen or weaken under high temperatures, diminishing the mechanical strength needed to bind together the active material, conducting agent, and current collector [23]. For instance, Zhang et al. reported that PAA cross-linked with hydroxyl propyl polyrotaxane demonstrates notable thermal stability for Si-based anodes. Polyimide (PI) stands out as a representative cyclic binder with high thermal stability, and various PI-based binders have been proposed (Figure 2c,d) [24]. Notably, fluorinated PI has been proposed as a binder with high thermal stability. The incorporation of heterocyclic imide rings and fluorine functional groups on the backbone of PI enhances its thermal stability [25]. Furthermore, Zhang et al. conducted a study comparing different binders in terms of their thermal diffusivity and thermal expansion. They deduced that the PAA binder exhibited the highest thermal diffusivity among the tested binders, with a value of 3.1 × 10^−^^3^ cm^2^ s^−^^1^, surpassing the values (1.0 × 10^−^^3^ and 9.1 × 10^−^^4^ cm^2^ s^−^^1^, respectively) for CMC and PVdF. The heightened thermal diffusivity of the PAA binder facilitates faster heat transfer to the electrode exterior, aiding in the dissipation of heat generated during battery operation and thereby enhancing battery stability [24]. Moreover, in the mixed state with active materials and conductive agents, the intra- and intermolecular interactions between polymer binders and other components can influence their secondary structures, significantly changing their thermal stability [26]. Therefore, the thermal stabilities of polymer binders are intricately linked not only to their primary and secondary structures but also to their kinetic motion, highlighting a need for systematic studies on the effects of key parameters on thermal stability.

#### 2.1.2. Electrochemical Stability

**Figure 3:**
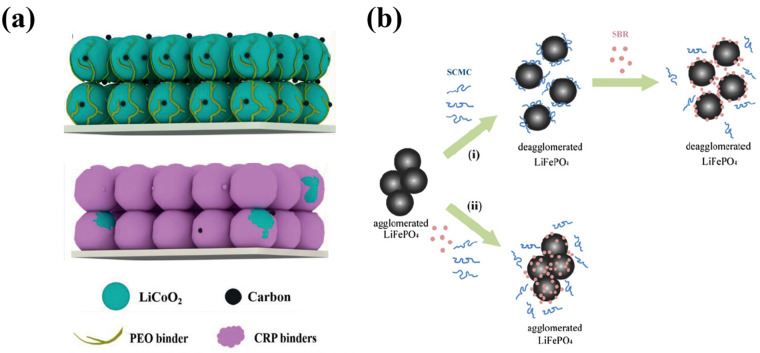
(**a**) Schematics for the binding capability/mechanism of PEO and CRP (carboxyl-rich polymer) binders. Reprinted with permission from [30], 2020, Wiley-VCH GmbH. (**b**) Dispersion mechanisms of LiFePO_4_ in an aqueous suspension in the presence of SBR and sodium carboxymethyl cellulose added via the sequences of (i) sequenced adding and (ii) the simultaneous adding process. Reprinted with permission from [37], 2012, Elsevier B.V.

Polymer binders undergo direct reduction or oxidation at low or high potentials, respectively, during the charge/discharge cycling of LIBs. Additionally, the decomposition of electrolytes on the active surfaces can generate active radicals that may react with polymer binders, resulting in their decomposition [5,27]. While conventional PVdF exhibits strong and symmetric C–F_2_ functional groups in a polyolefin structure, providing relatively high oxidation/reduction stabilities, its halogen bonding group lacks sufficient intermolecular interaction with active materials or conductive agents on their surfaces [28,29]. This deficiency leads to poor passivation behaviors for the active surfaces, causing significant electrolyte decomposition on both cathodes and anodes and contributing to poor cycling stability. To mitigate this issue, carboxyl-rich polymer (CRP) binders have been employed in conjunction with typical polyethylene oxide (PEO) and PVdF binders (Figure 3a) [30,31,32]. The CRP fully coats the active cathode surfaces, facilitating the passivation of the electrode surfaces. A similar passivation strategy is applied using a lithium polyacrylate (LiPAA) binder. Additionally, polymer binders with anti-oxidation properties are introduced onto active cathode surfaces through in situ cross-linking reactions [33]. A natural sericin binder has also demonstrated high voltage stability [34]. On the anode side, more severe electrolyte decomposition occurs owing to the substantial volume expansion of Si during charge/discharge cycling [35]. Consequently, polymer binders with multiple hydrogen bonding sites and high stretchability have been designed to fully coat active anode materials and accommodate the large volume change [30]. Interfacial degradation, involving electrolyte decomposition problems on the surface of the active material, can hinder the long-term cycling performance of the electrode. Various strategies have been studied to address this issue, including the use of electrolyte additives, radical scavengers, and the application of protective layers. Both inorganic and organic materials have been employed as protective layers to prevent direct contact between the electrolyte and active materials. Despite these efforts, many of these approaches face limitations because achieving complete coverage of the active particle surface with a protective layer is currently technically challenging. Moreover, even if complete coverage is achieved, it often results in increased interfacial resistance. Therefore, the binder must protect the surface of the active material while ensuring cohesion and adhesion between the particles and current collector. In a study conducted by Chang et al., λ-carrageenan (CRN), a sulfated polysaccharide, was employed as a binder to address interfacial degradation associated with electrolyte decomposition on the surface of the LiNi_0.5_Mn_1.5_O_4_ active material. The results demonstrated improved cycling and speed performance compared to conventional electrodes. The hydrophilicity of CRN enables it to cover the surface of active material particles uniformly, and the oxidative decomposition of the sulfate groups leads to the formation of a CEI/binder complex layer containing LiSO_x_F. This complex layer facilitates Li-ion conduction [36].

#### 2.1.3. Chemical Stability

Chemical stability is a critical requirement for polymer binders to prevent corrosion or decomposition under the operating conditions of a battery. Even in the case of PVdF binders known for their high chemical stability, an exothermic reaction between the C–F bond and lithiated carbon can form LiF [28,29]. Additionally, LiOH generated by residual impurities, such as water, can react with PVdF to produce LiF [36]. While byproducts resulting from the chemical instability of binders can be utilized as components of the SEI layer on the electrode surface, excessive accumulation can reduce the Coulombic efficiency (CE) and contribute to poor cycling stability through electrode collapse [38]. In certain cases, the chemical reactivity of binders has been harnessed for electrode fabrication. Binder precursors are mixed with active electrode materials, and in situ polymerization is initiated through various methods such as ultraviolet (UV) irradiation or heating [39,40,41,42]. This in situ polymerization enhances the adhesion properties of binders toward active materials through a mechanical interlocking effect or the formation of robust crosslinked networks [40,41,42].

The solubility of binders in the electrolyte significantly affects battery performance. PVdF, for example, exhibits swelling in organic solvents such as ethylene carbonate, diethyl carbonate, and dimethyl carbonate [43]. While the swelling phenomenon of binders in electrolytes can contribute to an improvement in ionic conductivity across binders, excessively high solubility that leads to the dissolution of binders in the electrolyte may have a detrimental effect on the integrity of the electrode [44].

#### 2.1.4. Dispersion Stability

The dispersion state of electrode materials, including the polymer binder in the slurry, plays a crucial role in ensuring the uniformity of electrode components. An uneven coating of active material, conductive additives, and polymer binder can adversely affect electron/ion conductivity, potentially leading to local current overloads and increased charge transfer resistances (R_ct_) [45,46]. In the slurry process, the polymer binder serves as a dispersant to distribute active materials and conductive additives stably [47]. The amphiphilic properties of the binder and its strong interactions with other components are key factors in achieving proper slurry viscosity and uniform coating.

Studies by Gordon et al. evaluated the impact of CMC and a fluorine/acrylate hybrid polymer (FAHP) on the dispersion of LiFePO_4_ (LFP) and carbon black particles in an aqueous slurry. CMC acted as a dispersant, influencing slurry viscosity to achieve effective particle dispersion in the manufacturing process, whereas the FAHP binder improved adhesion to the current collector [48]. Li et al. delved into the interaction between LFP, carbon-based conductive agents, and the CMC/SBR binder in an aqueous slurry. Similar to Gordon et al.’s findings, CMC played a role as a dispersant for the LFP particles in the aqueous system, with LFP preferring interaction with SBR, resulting in complementary binding behaviors. The enhanced dispersibility of the LFP particles with CMC was attributed to the electrostatic repulsion induced by an increased negative charge density, with the electrostatic force serving as a crucial factor for achieving the dispersion stability of the electrode materials (Figure 3b) [37].

Furthermore, slurry properties and preparation techniques notably impact electrode morphology, influencing electrochemical performance. Research by Kraytsberg et al. highlighted the relationship between shear stress applied by a mixer and cluster size in slurries. Notably, specific slurry techniques can modify the structure of the electrode components, leading to diverse electrochemical performance [49]. Consequently, changes in the binder material, solvent systems, or specific slurry techniques can affect the dispersion properties of the electrode materials in the slurry. Thus, a tailored engineering process is essential to establish an optimal dispersion state for electrode fabrication.

### 2.2. Mechanical Properties

The mechanical properties of binders, encompassing factors such as adhesive force, tensile strength, elasticity, and flexibility, are crucial considerations for their performance in LIBs. These properties directly impact the electrode fabrication process and the cycle life of LIBs, making it essential to select binders with specific mechanical attributes for the performance optimization of the LIBs [50].

Ideally, a binder should possess high adhesion, mechanical strength, elasticity, and flexibility. However, achieving a balance among these properties is challenging, particularly in the wet state under electrolyte soaking [51]. Additionally, pursuing good mechanical properties in polymers often involves promoting intramolecular interaction or increasing crystallinity [52]. While these characteristics enhance the mechanical strength of the polymer, they may have a downside, deteriorating the intermolecular interactions between the polymer and active material or conductive additive [53]. This, in turn, can adversely affect the electrode’s stability. Therefore, striking the right balance between the desirable mechanical properties and maintaining effective interactions with other components is a critical aspect of binder selection for LIBs [54]. The optimization of these properties not only ensures the successful fabrication of electrodes but also contributes to the overall stability and performance of LIBs throughout their cycle life.

#### 2.2.1. Adhesion

Adhesive force is a critical parameter extensively used to assess the performance of binders owing to its significant influence on the electrochemical performance of LIBs [46,51,55,56,57]. Binders with high adhesive force play a pivotal role in maintaining robust contact between the components of the electrodes, even amidst the volume expansion and contraction inherent in the charge/discharge cycle [58,59,60,61,62]. Several theories have been proposed to elucidate the mechanism of binder adhesion, including mechanical, chemical, and thermodynamic models [5,63]. The mechanical mechanism involves physical interlocking following the diffusion or penetration of the binder into the irregular or porous surfaces of the electrode materials. The effectiveness of mechanical interlocking is highly contingent on the surface roughness of the electrode materials [64]. Thermodynamic models describe adhesion through surface adsorption via van der Waals forces between two materials without the formation of chemical bonds [53]. The chemical model explains adhesion through the formation of surface chemical bonds, such as ionic, covalent, and hydrogen bonds [63,65]. To comprehensively describe the adhesion of polymer binders, these various mechanisms must be considered in combination with different circumstances. In general, the adhesion of low-polarity polymers, such as PVdF without nitrogen- or oxygen-containing functional groups, is primarily governed by mechanical interlocking and van der Waals forces [66]. However, polymers such as CMC, PAA, and alginate, which form robust chemical bonds such as hydrogen or ionic bonds, have demonstrated higher adhesive forces compared to PVdF [6,65]. The interaction between the binder and active material can also impact the bonding state during the wetting and drying processes. Techniques such as atomic force microscopy have been employed to study these interactions in detail [67]. Understanding and optimizing adhesive forces is crucial for enhancing the overall performance and durability of LIBs.

#### 2.2.2. Tensile Strength

The tensile strength of polymer-binder-based electrode materials indicates their maximum force resistance against mechanical failure and is closely tied to the structural stability facilitated by the polymer binder [68]. The tensile strength of polymers is significantly influenced by their chemical structure, molecular weight, and crystallinity. Generally, higher molecular weight, crystallinity, crosslinking density, and intermolecular interaction contribute to higher tensile strength in polymers [65,69]. In the context of LIBs, binders with high tensile strength offer advantages because they can endure repeated cycles of mechanical stress without undergoing deformation or failure [50]. This characteristic is particularly beneficial for polymer binders with high tensile strength, such as alginates and PI, which can mitigate the pulverization of Si-based anodes experiencing significant volume changes during battery operation [70,71,72,73]. However, it is important to note that polymers with high tensile strength may exhibit drawbacks, such as low ionic conductivity, which can diminish battery performance, or poor solubility, which can impede electrode processability. Striking a balance between tensile strength and other critical properties is essential for designing polymer binders that enhance the mechanical robustness of LIB electrodes while maintaining overall electrochemical performance [47,74,75,76].

#### 2.2.3. Flexibility and Elasticity

Elasticity and flexibility are crucial properties of binders, playing a significant role in maintaining a stable electrode structure during the volume changes that occur during battery operation [77,78]. Elasticity refers to the ability of a material to return to its original state after deformation, whereas flexibility describes the capacity of a material to bend without breaking. Binders with high elasticity and flexibility, typically characterized by polymers with low glass transition temperatures (T_g_), can effectively minimize electrode deformation caused by volume changes during charging and discharging cycles [79,80]. Physical or chemical crosslinking is a strategy that can dramatically enhance the elasticity of polymers, enabling them to endure mechanical deformation. For instance, physical crosslinking achieved through hydrogen bonding, pi–pi interactions, or host–guest interactions results in a three-dimensional network structure of polymer binders when mixed with active materials, resulting in significant mechanical robustness [81,82,83,84]. Chemical crosslinking, involving the formation of covalent bonds (e.g., ester bonds between PAA and poly(vinyl alcohol)(PVA)) or ionic bonds (e.g., alginate with calcium ions), enhances the cycling performance of LIBs [85,86,87,88]. These strategies improve the elasticity and flexibility of polymer binders, ensuring the structural integrity of electrodes over repeated charge and discharge cycles in LIBs.

### 2.3. Ionic Conductivity

The ion conductivity of the binder is a critical factor influencing the electrochemical performance of an LIB. When the binder uniformly and densely coats the active material, the impact of ion conductivity on the battery performance increases owing to the enhanced probability of ion transport through the binder [89]. Particularly for active materials with low ionic and electronic conductivity, such as olivine LFP, the introduction of polymer binders with high ionic conductivity can significantly enhance battery cycling performance [90,91]. In general, the ionic conductivity of polymers is closely associated with their T_g_. Below T_g_, polymers may exhibit poor ionic conductivity owing to limited chain mobility. Additionally, polymers with higher crystallinity generally exhibit low ionic conductivity owing to their reduced free volume for ion transport [79,80,92,93,94,95]. Consequently, strategies such as lowering the T_g_ or crystallinity are employed to enhance the ionic conductivity of polymers.

The affinity of the binder with the electrolyte and the wetting amount of the electrolyte are also crucial parameters influencing the ion conductivity of the binder [45,46]. While a higher wetting amount of the electrolyte can be advantageous for lithium-ion transport, excessive wetting can degrade the adhesive performance of the binder and the mechanical strength of the electrode [89]. Recent research has focused on improving the ion conductivity of binders to achieve high-performance LIBs. For example, the utilization of a binder system involving poly(3,4-ethylenedioxythiophene):poly(styrene sulfonate) (PEDOT:PSS) crosslinked by PEO with polyethyleneimine coating has demonstrated significantly higher electronic conductivity (~271 S cm^−^^1^) and a high ionic diffusion coefficient (4.0 × 10^−8^ cm^2^ s^−1^) compared to traditional binder systems such as CMC/acetylene black (~3 S cm^−^^1^ electronic conductivity and 2.8 × 10^−9^ cm^2^ s^−1^ ionic diffusion coefficient) for Si-based anodes [45]. Measurement techniques such as the galvanostatic intermittent titration technique (GITT) and electrochemical impedance spectroscopy (EIS) can be employed to determine the ion transport characteristics of the binder. GITT provides information on the lithium-ion diffusion coefficient, a crucial parameter for understanding the ion transport behavior of the binder. Higher lithium-ion diffusion coefficients indicate faster ion transport, contributing to better battery performance [96]. EIS analysis is valuable for evaluating the ion transport characteristics of the binder by measuring the resistance of the electrodes [90,97]. The measurement of electrode resistance using EIS enables the indirect identification of changes in lithium-ion movement during charge and discharge processes. The ohmic resistance of the electrode, primarily influenced by the binder, may increase owing to binder degradation or alterations in the electrode structure. Film resistance can also arise from the repetitive regeneration of the SEI layer, resulting in an elevation of the R_ct_ and a deterioration in battery performance. In summary, a comprehensive analysis of the ion transport characteristics of the binder is crucial for understanding its role in the electrochemical performance of the battery [89,91]. Achieving high ion conductivity and a high diffusion coefficient in the binder can contribute to improved battery performance; however, it is equally important to balance other factors, such as the adhesive performance and structural stability of the electrode [98].

## 3. Typical Binders

### 3.1. Anode Binders

#### 3.1.1. Polyvinylidene Fluoride (PVdF)

PVdF is a widely used polymer binder in secondary batteries, known for its thermal and electrochemical stability, making it suitable for various battery applications. However, when used as a binder for Si-based electrodes, PVdF shows certain limitations. One limitation of PVdF stems from its nonfunctional linear chain structure, which can result in low adhesive force and a weak interaction with Si-based materials, potentially leading to a degradation of battery performance [54]. Additionally, PVdF exhibits poor mechanical characteristics and flexibility, making it susceptible to the breakage of adhesive bonds during the volume changes that occur during the charging and discharging of Si-based electrodes, further impacting battery performance [77,78,89]. The nonreactive C-F structure of PVdF may also result in weak interactions between the electrode materials, hindering the formation of conducting channels [99,100]. The high viscosity of PVdF in solvents such as *N*-methyl-2-pyrrolidone (NMP) used in the slurry process can cause accumulation with the active material, potentially blocking charge-transfer pathways and degrading charge-transfer rates. However, researchers have demonstrated that adjusting the molecular weight of PVdF can influence its performance as a binder. Increasing the molecular weight of PVdF can lead to a higher binding strength and improved long-term cycling performance for the battery electrode [100].

Efforts have also been made to address the limitations of PVdF by developing composite materials. For example, synthesizing PVdF with other materials, such as lithium lanthanum titanate, has been studied to achieve improved conductivity for solid-state electrolytes [101]. In the broader context, it is crucial to carefully consider the characteristics of the polymer binder and select an appropriate one based on the specific requirements of the battery electrode and electrolyte system. Additionally, researchers are actively working on addressing environmental concerns associated with the use of solvents such as NMP in the slurry process and exploring more sustainable alternatives [102]. Understanding both the properties and limitations of PVdF, as well as other binder materials, is essential for designing high-performance batteries with improved electrochemical performance and environmental sustainability [6]. Ongoing research and development in this area will contribute to advancing battery technologies.

#### 3.1.2. Polyacrylic Acid (PAA)

PAA has emerged as a promising alternative binder to PVdF, particularly for electrodes subjected to substantial volume expansion, such as Si-based anodes [44]. PAA boasts several advantages, including applicability in a broad voltage range from graphite electrodes to Si anodes and solubility in both water and ethanol. This solubility feature reduces the reliance on toxic solvents such as NMP, contributing to its environmental friendliness [102]. The functional group of PAA contains carboxyl groups, imparting robust mechanical properties and facilitating interaction with Si particles through noncovalent bonding. This characteristic makes PAA well-suited for binding Si-based materials, particularly in scenarios where materials with severe volume expansion, such as Si anodes, are employed [103]. PAA has demonstrated versatility as a binder, finding application in replacing PVdF for alloy or conversion materials in batteries. To enhance the performance of PAA as a binder, ongoing research has explored innovative approaches [104]. For instance, Song’s group developed an interpenetrating gel polymer binder by creating a chemical structure bridge between PAA and PVA around Si particles. This network polymer binder exhibited exceptional cycle stability, highlighting a capacity retention of 1663 mA h g^−1^ after 300 cycles and a high CE of 99.3%. These advancements underscore the potential of PAA as a high-performance binder for Si anodes, offering solutions to some of the limitations associated with PVdF [86].

#### 3.1.3. Carboxymethyl Cellulose/Styrene Butadiene Rubber (CMC/SBR)

CMC is a linear polymer derivative of natural cellulose extensively studied as a binder for Si electrodes across various industries [105,106]. CMC, which is water-soluble, can establish robust hydrogen and covalent bonds with surfaces containing hydroxyl groups, such as Si. This unique property enables CMC to maintain high mechanical integrity within the battery cell without undergoing expansion in liquid electrolytes. In terms of its solubility, hardness, and elastic modulus in electrolyte solvents, CMC exhibits behavior akin to PAA [86]. To enhance the flexibility of CMC as a binder, researchers have studied combinations with other polymers, such as SBR. The utilization of a CMC/SBR mixed binder demonstrated an increase in the maximum elongation, leading to improved adhesion strength. Lee’s findings indicate that the maximum elongation and adhesive force are elevated while the tensile strength and modulus of the CMC/SBR binder may be slightly lower compared to PVdF. This mitigates the volume expansion of Si, resulting in significantly enhanced cycle performance, highlighting the benefits of employing CMC as a binder for Si electrodes [107]. Further studies, such as those conducted by He et al., have extended the application of CMC/SBR as an efficient binder for lithium–sulfur (Li-S) batteries, highlighting improved cycling performance with higher capacity retention compared to the PVdF binder. An electrode utilizing the CMC/SBR binder exhibited a capacity retention of 580 mA h g^−1^ after 60 cycles, outperforming the 370 mA h g^−1^ retention achieved using a PVdF binder. Additionally, it was noted that the anode using CMC/SBR exhibited lower resistance and charge transfer impedance, contributing to a more stable interface structure and an efficient electron transport network [108]. However, a challenge associated with binders such as PAA and CMC/SBR is the potential uneven coverage of the active material on the electrode surface, leading to localized mechanical stress and potential particle breakage [52]. Therefore, achieving uniform adhesion of the binder remains crucial, and ongoing research is dedicated to developing strategies to address this issue. Table 1 summarizes the advantages and disadvantages of the anode binders studied.

#### 3.1.4. Binders for Si/Graphite (Si/G) Anodes

The application of nickel-rich materials as cathodes in LIBs has garnered attention owing to their high energy density and cost effectiveness compared to their cobalt-based counterparts [4]. However, these Ni-rich cathodes often encounter challenges such as capacity retention issues and the development of an unstable cathode–electrolyte interface layer [109,110,111]. To address these concerns, extensive research is underway to enhance the performance of Ni-rich cathodes [112]. In parallel, researchers are exploring alternative anode materials to replace graphite, which has a limited theoretical capacity of 370 mA h g^−1^ [113,114,115,116]. Si has emerged as a promising anode material owing to its abundance, low operating voltage (~0.2 V vs. Li/Li^+^), and high theoretical capacity (3572 mA h g^−1^) [117,118]. Nevertheless, Si undergoes significant volume expansion (approximately 300%) during the intercalation/deintercalation process for lithium ions, in stark contrast to the volume expansion of graphite (approximately 10%). The substantial volume expansion of Si during cycling causes structural alterations in the electrode, leading to reduced adhesive forces between the substrate and active material, electrode peeling, and the formation of an unstable SEI layer [77,78]. These challenges may result in capacity decay, decreased CE, and heightened internal resistance in the electrode. Consequently, for Si to be successfully commercialized as a high-capacity anode material, it is imperative to restrict volume expansion to within 10% to minimize structural transformations, with a compression density of approximately 1.65 g cm^−3^. Moreover, the Si anode should exhibit a performance of ≥500 mA h g^−1^, achieve ~99% CE, and maintain ≥80% capacity retention even after 500 cycles to meet the criteria for large-scale energy storage systems [119].

Recently, both industry and academia have directed their research efforts toward designing Si anodes, employing a combination of Si and graphite along with suitable binders and electrolytes. This strategy aims to enhance the battery performance and tackle the inherent challenges associated with Si anodes [120,121,122,123]. The anticipated outcome of this approach is the development of LIBs characterized by high performance, cost effectiveness, and safety, making them well-suited to large-scale energy storage applications.

The compatibility between the polymer binder and electrolyte is a critical factor to consider. Luo et al. addressed this aspect by investigating the degree of swelling in the electrolyte based on the type of binder used [124]. A binder that allows for appropriate swelling in the electrolyte demonstrates a cooperative effect, enhancing lithium-ion movement and adhesion ability. Notably, the swelling ratio is influenced by factors such as temperature, salt concentration, and electrolyte composition. This observation underscores the significance of the mechanical properties of the swollen polymer, providing valuable insights into the stable performance of the battery [125].

Table 2 summarizes the electrochemical properties of Si/G composite materials, detailing the Si/G ratio, binder utilized, and electrolyte [47,74,75,76,99,100,126,127,128,129,130,131]. The data indicate that increased Si content leads to a higher capacity but lower CE and cycle retention. However, it is observed that the currently employed commercial binders and electrolytes may not be optimal for Si/G anodes, lacking the necessary stability during cycling for viable commercialization. This underscores the necessity for further research and development endeavors to identify and optimize suitable binders and electrolytes tailored specifically to Si/G composite anodes. This may involve the creation of novel binders or electrolytes designed to address the challenges associated with Si-based anodes, such as significant volume changes during cycling and the formation of an unstable SEI layer [105,106]. The quest for appropriate binders and electrolytes to ensure stable cycling performance for Si/G composite anodes is pivotal for successfully commercializing LIBs featuring Si-based anode materials. Continued research efforts in this domain can significantly contribute to the progress of Si/G composite anodes, addressing the challenges inherent in Si-based anode materials [97,132]. Ultimately, this research may bring us closer to realizing LIBs characterized by high capacity, low cost, and suitability for large-scale energy storage applications [131]. The challenges associated with the volume changes during the lithiation and delithiation of Si and graphite in Si/G composite anodes could impact the stability and cycling performance of LIBs. The substantial volume expansion of Si and the moderate volume expansion of graphite can induce mechanical stress, cracks, and the loss of electrical contact between the active materials and conducting agents [47,74,126,127,133]. These issues result in elevated internal resistance and irreversible cycling. Additionally, the repeated cracking and regeneration of the SEI layer during cycling may lead to the formation of an unstable and thick SEI, further compromising the battery’s performance and capacity. This phenomenon can also contribute to the consumption of lithium ions, leading to a gradual depletion of the usable electrolyte over time. To tackle these challenges, researchers are actively studying using different binder materials with diverse polymer characteristics in Si/G composite anodes [76,99,100,128,129,130]. The binder assumes a crucial role in upholding the structural integrity of the composite electrode, mitigating volume changes, and enhancing the cycling stability of the battery [77,78]. The meticulous selection and optimization of binder materials aims to minimize the stress and cracks induced by volume changes, enhance the electrical contact between active materials and conducting agents, and facilitate the formation of a stable and thin SEI layer [119,134]. This strategic approach can alleviate the issues of irreversible cycling and capacity fading linked with Si/G composite anodes, paving the way for stable cycling performance, even with higher Si content (up to 10%), in real-world commercial applications. The ongoing development of advanced binder materials and their effective integration into Si/G composite anodes remains a dynamic area of research [121,122]. Continued progress in this direction holds the potential to usher in the commercialization of high-performance LIBs featuring Si-based anode materials, effectively addressing the challenges associated with volume changes and SEI formation.

**Figure 4:**
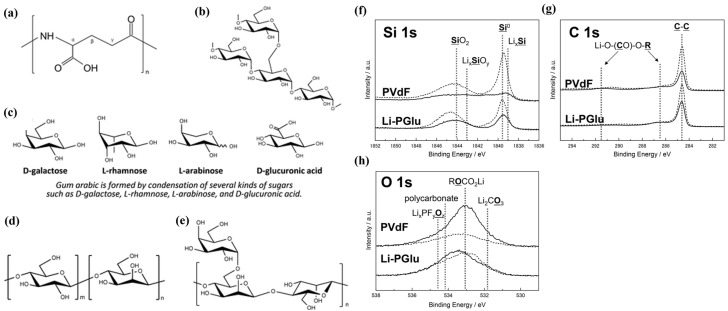
Molecular structures of natural polymers and monomers: (**a**) poly-y-glutamic acid; (**b**) amylopectin; (**c**) constituent monomers of gum arabic: d-galactose, L-rhamnose, L-arabinose, and D-glucuronic acid; (**d**) guar gum; and (**e**) glucomannan. HAXPES results for (**f**) Si 1 s, (**g**) C 1 s, and (**h**) O 1 s core-level spectra for Si/G composite electrodes with PVdF and Li-PGlu binders. Reprinted with permission from [100], 2017, American Chemical Society.

**Figure 5:**
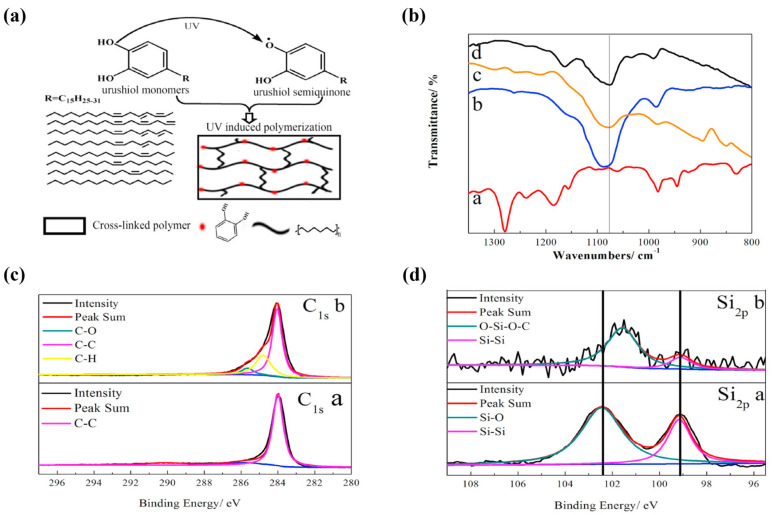
(**a**) Structure of urushiol and its UV curing mechanism. (**b**) FT-IR spectra for the expanded Si-O region (800–1300 cm^−1^) of urushiol monomers. (**c**,**d**) XPS Si 2p and C 1 s spectra for the Si/G powders and the powders scraped from the electrode with the Ur Binder. Reprinted with permission from [74], 2018, Elsevier.

Mochizuki et al. and Wang et al. conducted research focusing on enhancing the performance of Si/G composite electrodes in LIBs using different binder materials [100,135]. In their study, Mochizuki et al. employed lithium poly-γ-glutamate (Li-PGlu) and four natural polymers as binders in Si/G composite electrodes with a mass loading of 1 mg cm^−2^ [100]. The initial reversible capacities ranged from 800 to 1200 mA h g^−1^ (1.3 mA h cm^−2^), with a maximum CE of 51% to 79%. Li-PGlu emerged as an effective binder for suppressing electrolyte decomposition, providing uniform coverage of the active material surface. The binder was intentionally designed with a robust structure featuring polarized functional groups (-COOH and -NH-CO) (Figure 4a–e), promoting smooth lithium-ion movement in the electrolyte/electrode interface through coordination of the functional effects of oxygen and nitrogen atoms. The surface chemical and binding characteristics were confirmed using hard X-ray photoelectron spectroscopy (XPS), as shown in Figure 4f–h. Luo et al. utilized UV-cured urushiol monomers as binders in a Si/G composite electrode with a Si/G ratio of 90:10 and a mass loading of 0.8 mg cm^−2^ (Figure 5) [74]. The resulting electrode demonstrated a capacity of 603.3 mA h g^−1^ and an excellent capacity retention of 96.1% even after 400 cycles. The binder facilitated Si-O-C bond formation on the surface of the Si powder, and strong interaction between the surfaces of the Si particles and phenolic hydroxyl groups improved adhesion, limiting volume change and mitigating capacity loss and electrode degradation during volume expansion [119,134]. In another approach, Liu et al. synthesized a functional aqueous binder, PAA-vinyl triethoxy silane (VTEO), using lithium acrylate and VTEO. This binder was utilized in a composite electrode with a Si/G ratio of 97:3 and a mass loading of 0.8 mg cm^−2^ [75]. The PAA-VTEO binder exhibited a specific capacity of 470 mA h g^−1^ and a high cycle retention of 99% after 100 cycles. XPS analysis confirmed the formation of a strong 3D cross-linked network between the binder and silanol groups on the surface of Si nanoparticles, forming Si-OH groups and Si-O-Si covalent bonds. This highlighted the solid mechanical and binding properties of the PAA-VTEO binder, effectively mitigating volume expansion and enhancing electrochemical cycling stability. 

Each of these studies underscores the crucial role of binder materials in enhancing the performance and stability of Si/G composite electrodes in LIBs [119]. Binders possessing robust mechanical strength, elasticity, and effective adhesion to both Si and graphite particles are essential for suppressing the volume expansion of Si during cycling and maintaining the structural integrity of the electrode [74,75,126]. The careful selection and design of binders, considering appropriate functional groups and chemical characteristics, can significantly influence the electrochemical performance of composite electrodes. This impact extends to key aspects such as capacity retention, cycling stability, and the adhesion between active materials and conducting agents [99,100,128,129]. Continued research and the optimization of binder materials hold the potential to advance the development of high-performance Si/G composite electrodes for the batteries of the future. It is crucial to recognize that the interactions not only with Si but also with graphite play a pivotal role in determining the overall performance of Si/G composite electrodes [77,78]. Further exploration is necessary to comprehensively understand the intricate interactions between different binders and Si and graphite particles. This understanding will shed light on their effects on the mechanical and electrochemical properties of composite electrodes [97,132,136,137,138]. The cycle stability and overall performance of Si/G composite electrodes in advanced LIBs can be improved by meticulously optimizing binder selection and binder properties.

### 3.2. Cathode Binders

While extensive research has concentrated on developing high-performance cathode materials for LIBs, the significance of the binder in the cathode formulation should not be underestimated [6,91]. The binder assumes a vital role in upholding the structural integrity of the cathode electrode, ensuring effective adhesion between the active materials and the current collector and enhancing the overall electrochemical performance of the cathode [48]. Several challenges accompany the practical implementation of high-performance cathodes, including the detachment of active materials from the current collector, the dissolution of transition metal ions from the cathode materials, and the creation of an uneven SEI. These challenges can detrimentally impact the performance and cycle stability of LIBs [90,139]. Judicious selection of the binder can effectively address and overcome these issues. For instance, binders characterized by high adhesive strength and compatibility with both cathode materials and electrolytes can prevent the detachment of active materials from the current collector during cycling. Binders capable of adequately coating the cathode materials and forming a stable interface can impede the dissolution of transition metal ions from the cathode materials, thereby enhancing the long-term stability of the cathode [91]. Furthermore, binders with suitable mechanical properties play a crucial role in maintaining robust contact between the cathode and electrolyte, facilitating the development of a more uniform SEI and augmenting electrochemical performance. It is imperative to consider the type and crystal structure of the cathode material during binder selection because distinct cathode materials may impose varying requirements on the binder’s properties [48]. The binder should be meticulously chosen based on its compatibility with the cathode material, electrolyte, and other components of the battery, as well as its capability to address specific challenges associated with the cathode material. In conclusion, the selection of an appropriate binder is paramount for attaining high-performance LIBs, particularly in the cathode [90,140]. A judicious binder choice can effectively mitigate issues such as peeling, dissolution, and SEI formation, thereby contributing to enhanced cycle stability and the overall electrochemical performance of the cathode electrode. Ongoing research and developing advanced binders tailored to specific cathode materials and battery requirements are pivotal for advancing the next generation of LIBs [141].

#### 3.2.1. Binders for NCM

LiNi_1−x−y_Co_x_Mn_y_O_2_ (both x and y are ≤0.1, NCM) is a layered oxide under active research for application in high-energy LIBs. The performance of NCM cathodes can be improved by adjusting the ratio of the central atoms Ni, Co, and Mn, which directly influences capacity, performance, and safety [140]. Increasing the Ni content enhances the capacity of NCM cathodes. However, a significant challenge when using Ni-rich cathodes at high voltages is the choice of the binder material that holds the cathode together. PVdF is a commonly used binder for layered cathode materials; however, it has limitations when applied to high-voltage cathode materials, especially Ni-rich cathodes that are sensitive to moisture [142,143,144]. Exposure to water molecules can cause structural collapse and the corrosion of Ni-rich cathodes, producing alkaline chemical residues such as LiOH or Li_2_CO_3_. Therefore, there is a growing need for nonaqueous binders to replace PVdF and other aqueous binders in Ni-rich cathodes [6].

Recent research has shown promising results with the application of PI and an amphiphilic bottlebrush polymer (BBP) as alternative binders for NCM cathodes. Pham et al. demonstrated stable operation at high voltages using PI as a binder in NCM811 [140]. PI forms a robust binding environment through chemical bonding on the surface of NCM811, resulting in a high capacity of 203 mA h g^−1^ when charged up to 4.4 V. The strong interaction between -CF_3_ and PI bonds suppressed oxidation stability and metal dissolution, demonstrating the potential of PI as a binder for Ni-rich layered oxides. In another study, Kim et al. developed an amphiphilic BBP as a binder applicable to NCM811 cathodes [142]. This BBP integrated hydrophobic polynorbornene backbones with hydrophilic PAA sidechains. These hydrophilic PAA groups established robust hydrogen bonds with alkaline collectors, resulting in exceptional adhesion properties. In a 180° peel-off test, the adhesion force of the BBP binder with the aluminum current collector was quantified at 3.76 N cm^−1^, demonstrating a substantial improvement compared to the PVdF film (0.18 N). Remarkably, the BBP binder exhibited consistent cycling performance over 240 cycles at a high mass loading of 27 mg cm^−2^, with a minimal content of 1 wt%. Furthermore, it displayed electrochemical stability similar to the PVdF binder, underscoring its outstanding performance. This implies that nonaqueous solvent-based binders such as PI and the amphiphilic BBP hold potential for application in NCM811 cathodes, known for their nickel richness and the necessity for robust structural stability [140,142]. Further exploration and research into binder materials will significantly contribute to the advancement of high-performing LIBs, particularly those featuring nickel-rich cathodes [143].

#### 3.2.2. Binders for LFP

LFP, as a cathode material, exhibits a distinctive one-dimensional channel movement of lithium ions within its olivine crystal structure, enabling a theoretical capacity of 170 mA h g^−1^ through insertion/extraction processes [90,91]. Renowned for its robust covalent bonds and minimal volume alterations during charging and discharging, LFP exhibits remarkable stability, especially when compared to cathode materials with layered structures, such as NCM. Unlike anode materials such as Si, which undergo substantial volume expansion, LFP typically does not necessitate binders with high mechanical strength [145]. However, despite its advantages, LFP grapples with challenges associated with its low electrical conductivity (approximately 10^−9^ to 10^−10^ S cm^−1^) and Li^+^ ion diffusivity (approximately 10^−14^ to 10^−16^ cm^2^ s^−1^), factors that can impact its overall performance. Consequently, ongoing research is directed toward exploring low-cost, water-soluble, conductive binders tailored for LFP cathodes [146].

In a study by He et al., an environmentally friendly water-soluble binder referred to as xanthan gum (XG), a natural and nontoxic polysaccharide, was applied in cathodes with LFP [102]. Although XG has lower adhesive forces compared to the conventional PVdF binder, it possesses abundant functional groups such as carboxyl and hydroxyl, resulting in a higher slurry viscosity. The functional groups in XG may promote electron and ion conduction by providing more active binding sites between the LFP, conducting agents, and substrate and improving dispersion in the slurry manufacturing process. The XG binder exhibited excellent cycling stability and performance at high speeds, maintaining 55.3% of its capacity at 5 C speeds. The PVdF and CMC binders maintained 34.8% and 57.8% of their capacity, respectively. This suggests that XG has the potential to be a new water-soluble binder for LFP cathodes, offering advantages such as low cost, high viscosity at low concentrations, and excellent processability (Table 3).

## 4. Sustainable Binders for LIBs

### 4.1. Bio-Based Eco-Friendly Binders

The choice of an eco-friendly binder, replacing commonly used binders such as PVdF, is essential for the design of sustainable batteries. The environmental impact of battery disposal has become a significant concern, with conventional binders potentially releasing harmful products [135,147,148]. Therefore, interest is growing in researching natural binders based on biopolymers, aiming to develop sustainable battery technologies that minimize environmental impact throughout their lifecycle, including their disposal.

In a recent study, Yoon et al. utilized a biodegradable polymer, amorphous poly(3-hydroxybutyrate-*co*-4-hydroxybutyrate) (aPHA), as a binder for LIBs [149]. The chemical structure of the aPHA binder, containing 47% 4-hydroxybutyric acid in the monomer 3-hydroxybutyric acid, demonstrated favorable properties for electrode maintenance and smooth ion transfer. When compared to PVdF, even with a reduced amount of binder and increased active material, the aPHA binder maintained a capacity of 324 mA h g^−1^ and a CE of 94.1%, demonstrating its viability as an alternative binder owing to its biodegradable properties. Similarly, Nowak et al. employed a biodegradable polymer, poly(hydroxybutyrate-*co*-hydroxyvalerate) (PHBV), as a binder for a lithium-ion battery with a graphite anode [150]. Compared to the conventional binder PVdF, PHBV exhibited a similar specific capacity and lithium-ion diffusion coefficient in the graphite electrode. After 100 cycles, PHBV showed a specific capacity of 357 mA h g^−1^ and 99.1% capacity retention, highlighting its potential as a replacement binder for anodes in LIBs. These findings suggest that biopolymer-based binders have the potential to be the next-generation binders for LIBs, offering durability to the electrodes while promoting the development of sustainable batteries (Table 4).

### 4.2. Water-Based Process

The pursuit of sustainable manufacturing for LIBs has led to significant attention to water-soluble polymeric binders. Traditional binders such as PVdF often require specific organic solvents such as NMP in the LIB manufacturing process [5,151]. The use of PVdF dissolved in NMP during the slurry process has drawbacks, including a high boiling point, toxicity, flammability, cost, and environmental damage [151,152]. Research has focused on water-soluble binders to address these issues and eliminate the application of conventional PVdF binders and organic solvents [151,152,153]. Various water-soluble binders have been studied, including both natural and synthetic polymers. Natural polymers such as polysaccharides (CMC [105,106], carrageenan [154], alginate [70,88,155], chitosan [156,157], gums [152,158,159,160], cyclodextrins [161,162], gelatin [163,164,165], and lignin [166]) and synthetic polymers (PAA [86,97,132,136,137,138], PVA [42,86,167,168], polyacrylamides [11,12,169], PI [71,72,73], and SBR [43,153]) have been investigated for water-based processes in LIBs. These water-soluble binders typically contain hydrophilic polar functional groups such as hydroxyls, carboxylic acids, amides, and amines. These functional groups enhance water solubility and improve the adhesion strength to electrode materials by forming hydrogen bonds. Notably, while water-soluble binders such as CMC are often used in combination with SBR because of their brittle nature, the polar functional groups may reduce the binder’s flexibility through strong intramolecular interactions, increasing T_g_ and brittleness [43]. Additionally, while water-soluble polymeric binders have found success with carbonaceous anode materials, their application in cathode fabrication remains challenging owing to the hygroscopic degradation of the cathode active materials. While recent reports indicate success with non-hygroscopic cathode materials such as LFP and spinel-type LiMn_2_O_4_ using CMC binders, the hygroscopic nature of Ni-rich cathode materials, such as NCM, poses challenges for water-soluble polymers [145,170]. The high voltage and energy capabilities of Ni-rich cathode materials make them promising for LIBs; however, their susceptibility to water-induced degradation complicates the use of water-soluble polymers. Overcoming these challenges is crucial for advancing the application of water-soluble binders in the sustainable manufacturing of high-performance LIBs [143,144].

### 4.3. Dry Process and Ultra-Thick Electrodes

The ultimate objective of binder research is to facilitate the design of ultrathick electrodes to increase the energy density per weight of LIBs [139,171]. The conventional cell design involves stacking multiple layers of anodes and cathodes, each with a thickness of 15–25 μm. Researchers are evaluating ways to minimize inactive parts, such as current collectors and separators, by increasing the thickness of the active material in the anode and cathode to 200 μm or more. This approach aims to reduce dead volume, increase energy density, and potentially lower manufacturing costs by eliminating the need to assemble multiple layers [15,16,17,172,173]. Empirical investigations have demonstrated that augmenting the electrode thickness from 70 to 320 μm can yield a substantial 19% enhancement in the volume energy density [13]. While increasing the electrode thickness has shown promise in increasing the volume energy density, challenges can arise, including crack generation during drying and the fragile mechanical properties of thick electrodes [174]. Additionally, thicker electrodes face increased charge transport distance and resistance, making it challenging to achieve electrochemical performance comparable to standard-thickness electrodes [9,10,175]. To overcome these challenges, various strategies, such as electrode designs to promote ion transport and electrode designs with low tortuosity, have been explored. However, using a suitable binder with excellent interfacial adhesive forces between the anode and cathode materials is a promising solution for designing high-energy-density batteries with thick electrodes. This would enable strong adhesion with only a small amount of binder, addressing the challenges associated with thick electrodes. Recent research has focused on dry-coating processes as an efficient production method for thick electrodes without using solvents [176]. Dry-coating processes eliminate the need for toxic organic solvents, facilitating the manufacture of high-loading electrodes with increased active materials [177,178]. The binder plays a crucial role in providing adhesive forces between particles within the electrode and forming a network of particles, whether in dry- or wet-coating processes. In wet-coating processes, conductive materials and binders with a relatively low density can rise above the electrode during solvent evaporation, resulting in nonuniform distribution [179,180,181]. By contrast, dry-coating processes, which do not involve solvent evaporation, allow for a uniform distribution of electrode materials even in thick electrodes. Micro-computed tomography observations conducted by Ryu et al. confirmed that dry-coating electrodes form a denser and more continuous conductive network. In wet-coating electrodes, the binder undergoes dissolution in a solvent, encasing the active material particles [178]. Conversely, in dry-coating electrodes, the binder and agglomerates of conductive materials are interspersed among the active material particles, covering only a fraction of their surface. This differentiation allows dry-coating electrodes to expedite ion transfer, ultimately augmenting electrochemical performance [14,182].

**Figure 6:**
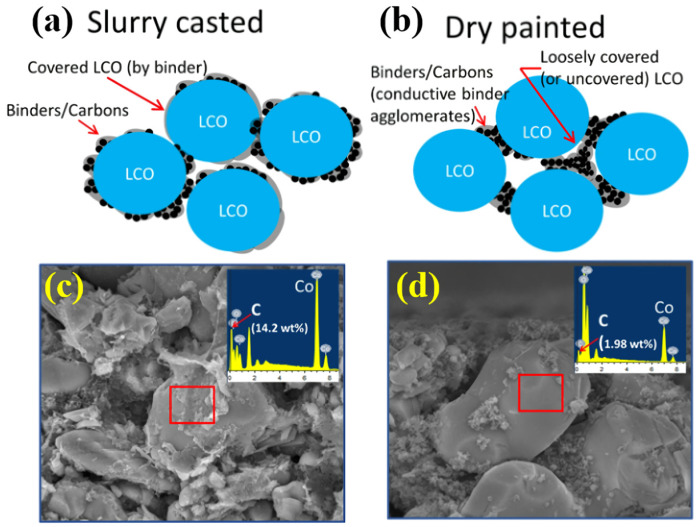
Schematics of characteristic binder/carbon distributions in (**a**) dry-coating electrodes and (**b**) wet-coating electrodes. SEM images showing representative LiCoO_2_ particles in cross-sectioned (**c**) dry-coating electrodes and (**d**) wet-coating electrodes. Reprinted with permission from [182], 2016, Springer Nature Limited.

In contrast to wet-coating electrodes, which rely on a solvent, dry-coating electrodes rely on particle cohesion established through surface energy. Consequently, the particle distribution in dry-coating electrodes is dictated by interparticle bonding arising from surface energy rather than solvent effects (Figure 6). This results in a cohesive interaction between the binder and the active material, surpassing the cohesion between the binder and the conductive material. Consequently, agglomerates composed of the binder and the conductive material are formed between the active material particles [182]. Establishing a well-connected network between the active materials through the binder is particularly crucial in dry-coating electrodes. Li et al. introduced a hot-press-based method to manufacture thick dry-coating electrodes by affixing electrode particles onto a current collector [51]. Furthermore, they achieved a robust adhesive force between the active material and the current collector in the electrode by employing phenoxy resin as a binder. EIS measurements revealed an R_ct_ value of 40.15 Ω for the dry-coating electrode using phenoxy resin as a binder, in contrast to 44.06 Ω for the dry-coating electrode using PVdF as a binder, which exhibited lower resistance to charge transfer. Additionally, cyclic voltammetry measurements demonstrated that phenoxy resin exhibited electrochemical stability within the operating voltage range and was deemed suitable as a binder for thick electrodes. The dry-coating electrode produced using phenoxy resin (~40 mg cm^−2^) displayed stable cycling performance for 50 cycles at 0.1 C [14]. In applying the dry-coating method to electrode manufacturing, achieving a uniform distribution of electrode powders is feasible, thereby enhancing cycle stability.

## 5. Advanced Battery Systems

### 5.1. Li-Air Battery

Li-air batteries (LABs) rely on the reversible formation and decomposition of cathode materials, boasting a substantial theoretical specific energy of 3600 Wh kg^−1^, positioning them as a promising contender for future energy storage systems. The pivotal reactions within LABs, the oxygen evolution reaction and the oxygen reduction reaction, occur on the cathode’s surface. Therefore, a cathode with a larger surface area holds an advantage in achieving a high discharge capacity. Moreover, a cathode with an ample surface area facilitates the absorption of electrolytes and helps prevent pore clogging caused by the formation of Li_2_O_2_, a byproduct [183]. Choosing an appropriate binder is crucial in LABs. The chemical stability of the binder is constrained by the Li_2_O_2_ produced through the elution of LiO_2_ in a competitive reaction involving superoxide radicals and the binder [184]. Although LABs commonly employ PVdF binders, their chemical dehydrofluorination can reduce their capacity or induce overvoltage [185]. Nasybulin et al. evaluated the stability of 11 different polymer binders, including PVdF, against reduced oxygen species generated during the discharge processes of LABs. While most polymers exhibited decomposition properties in the LAB operating environment, polyethylene (PE) demonstrated excellent stability against superoxide and peroxide species. Consequently, PE emerged as a robust binder suitable for the prolonged operation of LABs [186].

When porous carbon is utilized in a LAB cathode, a challenge arises with decreased cycle efficiency owing to side reactions, such as the formation of Li_2_CO_3_. The application of a carbon-free electrode in the cathode proves advantageous, suppressing side reactions caused by carbon and ensuring a lifespan exceeding 100 cycles. Carbon-free electrodes predominantly employ metal nanowires, where the role of a binder becomes pivotal in maintaining the nanowire structure. Jung et al. employed a porous carbon-free conducting nanopaper using Ag nanowire (AgNW) as the anode. In this case, chitin, an eco-friendly biopolymer with robust bonding properties, served as the binder, preserving the framework of AgNW. Unlike the PVdF binder, prone to decomposition in the air cathode, and the poly(methyl methacrylate) binder, characterized by low adhesion, the chitin binder forms a mechanically resilient conductive framework through strong adhesion and chemical stability [187].

### 5.2. Li-S Battery

Li-S batteries have garnered significant attention owing to the impressive theoretical capacity (1672 mA h g^−1^) and the energy density (2600 Wh kg^−1^) of sulfur. However, their potential is hindered by kinetic challenges arising from sulfur’s low intrinsic electronic/ionic insulation and significant volume change of up to 80% during cycling [188]. A major concern with Li-S batteries is the dissolution of lithium polysulfide intermediates (Li_2_S_n_) into the electrolyte during cycling, generating polysulfide byproducts that contribute to low CE and cycle stability. Given these challenges, commercialization of Li-S batteries for practical use remains limited. Numerous strategies have been explored to address these issues [189]. Specifically, binder research on Li-S batteries aims to develop efficient poly binders capable of capturing intermediate polysulfide species and preventing the loss of active materials from the sulfur cathode. The conventional binder used in Li-S batteries, PVdF, lacks affinity for intermediate polysulfides and functions merely as a basic bond. Recent advancements include the development of dual-functional polymer binders, exemplified by Li et al.’s study [190]. They utilized Li^+^-Nafion and polyvinylpyrrolidone (PVP) as binders in a Li-S battery. PVP, an oxygen-rich polymer, demonstrated superior polysulfide absorption capacity compared to PVdF, whereas Li^+^-Nafion effectively inhibited the polysulfide shuttle by impeding polysulfide transport. In a separate study, Liu et al. enhanced cycle stability in Li-S batteries by incorporating Cu^2+^ ions, serving as an ionic crosslinker, into a sodium alginate binder. The addition of Cu^2+^ ions proved effective in fixing the polysulfide, further contributing to improved cycle stability [191].

### 5.3. Solid-State Battery

LIBs typically utilize a highly flammable liquid electrolyte, raising safety concerns because of the potential for fire or explosion. Solid-state lithium batteries (SSLBs) have emerged as promising alternatives to LIBs, offering the advantage of maintaining the electrode’s shape over extended cycles. Similar to LIBs, SSLBs require the selection of an appropriate polymer binder to prevent delamination between the cathode components (active material, conductive carbon, and solid electrolyte) and to uphold a stable electrode structure during cycling [192]. Research by Oh et al. has demonstrated effective cycle stability in sulfide-based all-solid-state batteries employing an elastic polymer known as “Spandex” as a binder [193]. This choice effectively buffered volume changes occurring during charging and discharging. Herein, the hard segments, consisting of urethane, methylene diphenyl diisocyanate, urea, and ethylene diamine, formed robust hydrogen bonds with hydroxide groups on the particle surface, imparting a high bonding strength within the electrode. Meanwhile, the soft segments, comprised of poly(ethylene glycol), provided elasticity through stretching–unstretching motion, serving to buffer volume changes during charging and discharging. This contributed to improved mechanical stability and long-term cyclability in the electrode. Jin et al. introduced a crosslinked binder network through in situ polymerization, enhancing cathode interaction and preventing electrode particle exfoliation. The terpolymer underwent amidation with polyethyleneimine, forming a mild cross-linked binder network at ambient temperature. Subsequent curing at 120 °C strengthened the cross-linking density for a robust electrode framework [194].

## 6. Conclusions and Outlook

This review has emphasized the critical characteristics that binders should possess for their application in LIBs, catering to both anode and cathode materials. Numerous factors come into play when choosing a polymer binder, including electrochemical stability, thermal stability, compatibility with electrolytes, solubility in solvents, mechanical properties, ion conductivity, and dispersion stability. For anode materials, binders must exhibit excellent mechanical properties and elasticity to uphold the structural integrity of the electrode, especially with materials such as Si that undergo substantial volume changes. Understanding the interaction mechanisms with both graphite and Si surfaces is crucial for effective binder selection. The choice of binder for cathode materials varies depending on the crystal structure of the cathode material. Different polymers, such as aqueous and nonaqueous binders and conductive polymers, may be suitable for different cathode materials. Additional considerations in binder design include cost effectiveness, strong adhesion, ease of processing, and eco-friendly properties. Adopting low-cost, environmentally friendly, and biodegradable polymers contributes to sustainable development and helps mitigate the environmental impact of batteries, even after disposal. This review serves as a valuable reference for understanding fundamental requirements in binder design for high-performance LIBs, offering insights into the selection of appropriate binders for various electrode materials, particularly in the context of thick electrodes. The principles and findings established in this review can extend to other advanced battery systems, such as Li-air, Li-S, and solid-state batteries. This paves the way for the development of next-generation batteries that not only exhibit improved performance but also adhere to sustainability principles.

## Figures and Tables

**Table 1:** Advantages and disadvantages of anode binders.

Binder	Advantages	Disadvantages	Ref.
PVDF	- Thermal stability - Electrochemical stability	- Poor mechanical characteristics - Low flexibility	[54,77,78,89]
PAA	- Broad voltage range - Solubility in water and ethanol - Robust mechanical properties - Facilitates interaction with Si particles	- Uneven cover of the active material - Localized mechanical stress - Potential particle breakage	[52,103]
CMC/SBR	- High elongation and adhesive force - High mechanical integrity - Robust hydrogen and covalent bonds with Si surfaces	- Uneven cover of the active material - Localized mechanical stress - Potential particle breakage	[52,86]

**Table 2:** Electrochemical properties of various Si/G electrodes according to their Si/G ratios and different types of binders and electrolytes.

Si/G Ratio	Binder *	Electrolyte	CE (%)	Cycle	Ref.
Si/G (10:90)	Ur	1 M LiPF_6_ EC/EMC/DMC (1:1:1) (3 wt% FEC)	99.6	400	[74]
Si/G (2.5:97.5)	PAA-VTEO	1 M LiPF_6_ EC/DMC (1:1) (3 wt% FEC)	89.4	477	[75]
Si/G (50:50)	reDNA/ALG	1 M LiPF_6_ EC/DEC (1:1) (5 wt% FEC)	99.1~99.6	300	[126]
Si/G (19:57)	*c*-Alg-*g*-PAAm	1.15 M LiPF_6_ EC/DEC/DMC (3:5:2) (5 wt% FEC, 2 wt% VC, 5 wt% LiBF_4_)	72.8	100	[127]
Si/G (15:73)	SSC4SA	1 M LiPF_6_ EC/DEC/FEC (1:1:0.2)	99	200	[47]
Si/G (15:73)	GC-*g*-LiPAA	1.2 M LiPF_6_ EC/DMC (3:7) (10 wt% FEC)	90.3	100	[76]
Si/G (19:57)	Alg-*g*-PAMAT	1.5 M LiPF_6_ EC/DEC/DMC (3:5:2) (5 wt% FEC, 2 wt% VC, 0.4 wt% LiBF_4_)	56~62 (capacity retention)	200	[128]
Si/G (43:43)	PAA	1 M LiPF_6_ DMC/FEC (7:3)	88~91	40	[99]
Si/G (30:50)	Li-PGlu	1 M LiPF_6_ EC/DMC (1:1) (2 v% FEC)	73	30	[100]
Si/G (15:73)	CMC/SBR = 4:6(*w*/*w*)	1.2 M LiPF_6_ EC/DEC (3:7) (30 wt% FEC)	99.8~99.9	400	[129]
Si/G (15:73)	LiPAA	1.2 M LiPF_6_ EC/EMC (3:7) (10 wt% FEC)	91	50	[130]
Si/G (20:65)	LiPAA	1 M LiPF_6_ EC/DEC/FEC(3:6:1)	79.1	50	[131]

* Ur: urushiol, re-DNA/ALG: renatured deoxyribonucleic acid (reDNA) and alginate (ALG), *c*-Alg-*g*-PAAm: complete alginate grafted with polyacrylamide, SSC4SA: poly(N-methylperfluorobutane-1-sulfonamide ethyl acrylate), GC-*g*-LiPAA: glycol chitosan-graft-lithium polyacrylate, Alg-*g*-PAMAT: alginate-graft-poly(acrylamide-co-acrylamide aniline tetramer), EC: ethylene carbonate, EMC: ethyl methyl carbonate, DEC: diethyl carbonate, DMC: dimethyl carbonate, FEC: fluoroethylene carbonate, VC: vinylene carbonate.

**Table 3:** Comparison of the electrochemical performances of cathodes with different binders at a C/5 rate.

Cathode	Binder	Specific Capacity (mA h g^−1^, 100th)	Capacity Retention (%, 100th)	Ref.
NCM	PI	160.37	91	[140]
	PVdF	52.5	74	
LFP	XG	151.1	96.9	[102]
	CMC	151.2	97.6	
	PVdF	151.6	92.8	

**Table 4:** Comparison of two biodegradable polymer binders.

Binder	Electrode	Specific Capacity (mA h g^−1^, 100th)	Ref.
aPHA	Graphite: Carbon black: aPHA = 8:1:1	324	[149]
PHBV	Graphite: Carbon black: PHBV = 8:1:1	357	[150]
